# The impact of alpha-fetoprotein (AFP), child-turcotte-pugh (CTP) score and disease staging on the survival of hepatocellular carcinoma (HCC) patients: a retrospective cohort from single oncology center

**DOI:** 10.32604/or.2024.050903

**Published:** 2024-12-20

**Authors:** NASSER MULLA, YOUSEF KATIB, ASIM M. ALMUGHAMSI, DUAA S. ALKHAYAT, MOHAMED MOSAAD, SAMIR T. ALFOTIH, RAWAN ALAOFI

**Affiliations:** 1 Department of Internal Medicine, Faculty of Medicine, Taibah University, Madinah, 42278, Saudi Arabia; 2 Department of Radiology, Faculty of Medicine, Taibah University, Madinah, 42278, Saudi Arabia; 3 Department of Surgery, Faculty of Medicine, Taibah University, Madinah, 42278, Saudi Arabia; 4 Department of Endemic and Infectious Diseases, Suez Canal University, Ismailia, 41522, Egypt; 5 King Salman bin Abdulaziz Medical City, Madinah, 42311, Saudi Arabia; 6 Prince Mohammed Bin Abdulaziz Hospital, Madinah, 41511, Saudi Arabia

**Keywords:** Hepatocellular carcinoma, Pattern, Alpha-fetoprotein (AFP), Child-turcotte-pugh, Progression free survival

## Abstract

**Background:**

Hepatocellular carcinoma (HCC) is the most common cause of cancer-related death in Saudi Arabia. Our study aimed to investigate the patterns of HCC and the effect of TNM staging, Alfa-fetoprotein (AFP), and Child-Turcotte Pugh (CTP) on patients’ overall survival (OS).

**Methods:**

A retrospective analysis was conducted on 43 HCC patients at a single oncology center in Saudi Arabia from 2015 to 2020. All patients had to fulfill one of the following criteria: (a) a liver lesion reported as definitive HCC on dynamic imaging and/or (b) a biopsy-confirmed diagnosis.

**Results:**

The mean patient age of all HCC cases was 66.8 with a male-to-female ratio of 3.3:1. All patients were stratified into two groups: viral HCC (n = 22, 51%) and non-viral HCC (n = 21, 49%). Among viral-HCC patients, 55% were due to HBV and 45% due to HCV. Cirrhosis was diagnosed in 79% of cases. Age and sex did not significantly statistically differ in OS among viral and non-viral HCC patients (*p*-value > 0.05). About 65% of patients had tumor size >5 cm during the diagnosis, with a significant statistical difference in OS (*p*-value = 0.027). AFP was >400 ng/ml in 45% of the patients. There was a statistically significant difference in the OS in terms of AFP levels (*p*-value = 0.021). A statistically significant difference was also observed between the CTP score and OS (*p*-value = 0.02). CTP class B had the longest survival. BSC was the most common treatment provided to HCC patients followed by sorafenib therapy. There was a significant statistical difference in OS among viral and non-viral HCC patients (*p*-value = 0.008).

**Conclusions:**

The most common predictors for OS were the underlying cause of HCC, AFP, and tumor size. Being having non-viral etiology, a tumor size >5 cm, an AFP > 400 ng/mL, and a CTP score class C were all negatively associated with OS.

## Introduction

Hepatocellular carcinoma (HCC) ranks as the fifth most prevalent cancer globally, with 900 thousand new cases identified in 2020 [1]. The occurrence of HCC in developing nations is three times greater than in Western countries. Despite significant advancements in HCC management, overall outcomes remain suboptimal [1,2]. HCC is a significant health burden in Saudi Arabia. According to the 2021 Saudi Cancer Registry, HCC is the ninth most common cancer overall, ranking sixth among males and twelfth among females. It is also the most common cause of cancer-related deaths in Saudi Arabia [3]. In 2019, King Faisal Specialist Hospital and Research Center in Saudi Arabia reported HCC as the fourth most common malignancy among males in the hospital [4]. Madinah city is considered the third reported city for HCC in Saudi Arabia with a high incidence rate of 6.6 per 100,000 in 2016 [5].

The Saudi Association for the Study of Liver Diseases and Transplantation (SASLT) established a multi-disciplinary task force to enhance guidelines previously issued by the Saudi Gastroenterology Association [5]. Identifying risk factors for HCC and implementing appropriate strategies for screening high-risk populations are essential for early detection and improved prognosis. Liver cirrhosis stands as the most prevalent risk factor for HCC [6], with an annual incidence of approximately 3% in cirrhotic patients [6]. Around 80% of these cases are linked to viral causes [7], although this percentage varies geographically and is influenced by the prevalence of hepatitis B virus (HBV) and hepatitis C virus (HCV) [8,9]. It can also be categorized into cirrhosis-related and non-cirrhosis-related. Cirrhosis-related factors encompass HBV or HCV infection, alcoholic cirrhosis, genetic mutations, non-alcoholic fatty liver disease (NAFLD), primary biliary cholangitis, and alpha-1 antitrypsin deficiency [8]. HBV is the leading cause of HCC in East Asia and Africa while HCV is the leading cause in Western countries [10]. In Saudi Arabia, 80% of HCC cases are associated with HBV or HCV infection [11–14]. Recently, NAFLD-related HCC has also attracted more attention since a growing population worldwide is estimated to have NAFLD [15].

The diagnosis of HCC is based on a combination of clinical and laboratory features as well as imaging and pathological biopsy appearance. However, the final diagnosis is always based on imaging techniques or tissue biopsy. In the advanced stage, patients may have typical symptoms including right hypochondriac pain, jaundice, ascites, and liver failure [16]. However, many patients are asymptomatic in the early stages. The early stage has potential curative treatments including surgical resection, radiofrequency ablation (RFA), and liver transplantation (LT) [17]. However, most patients are diagnosed at an advanced stage that delays effective therapeutic interventions and carries a poor OS [18,19] Ultrasound (US) is the most widely recommended method for HCC diagnosis [20]. It determines the size-based pathway especially if high-risk patients present with a nodule or a mass in the liver. Previous studies found that small nodules (<1 cm) were unlikely to be HCC nodules [21,22]. Liver nodules larger than 1 cm in size should undergo evaluation using dynamic contrast-enhanced computed tomography (CT) and/or magnetic resonance imaging (MRI). For patients who do not exhibit classic imaging and serology for HCC, a needle biopsy of a suspicious lesion is necessary [22].

Alpha-fetoprotein (AFP) is recognized as a clinical screening biomarker for HCC; however, its diagnostic utility is still under investigation. Elevated serum AFP levels typically indicate a heightened risk of HCC development and a poor prognosis [23,24]. During the early stages of hepatocyte malignant transformation, the AFP gene becomes activated within the cell, leading to increased gene expression [24,25]. The European Association for the Study of the Liver (EASL) guideline characterizes AFP as a suboptimal screening test due to its susceptibility to interference from viral replications and the underlying liver disease type [26]. Several studies indicated that AFP is a non-sensitive and non-specific screening biomarker for HCC as its elevation may be found in less than 20% of patients with early HCC [27,28]. However, using AFP in the US may aid in the early diagnosis of HCC although early detection of AFP and cancer are not associated with better outcomes. A single study about the use of AFP in HCC was conducted in Saudi Arabia [29]. The sensitivity of AFP in the detection of HCC in 206 Saudi patients was estimated to be 60%–75%. The study concluded that AFP had a poor diagnostic value for HCC. Lesion less than 1 cm, repeated US every 3 months is recommended [5]. The high-risk population varies according to the guidelines which include patients with chronic HBV/HCV or liver cirrhosis [1]. Saudi Arabian Guidelines added a Child-Turcotte Pugh (CTP) classification into consideration. The Saudi Guidelines suggest investigation of all cirrhotic patients, but it also stated that there was insufficient evidence to advise surveillance for patients with HCV without cirrhosis [1].

The treatment algorithm for HCC is dynamic and subject to frequent updates. Updated guidelines typically evaluate surgical and non-surgical approaches in the context of HCC management. Prognostic outcomes are heavily influenced by HCC staging and treatment selection. Various staging systems are employed to determine the most appropriate treatment modality for HCC patients. Commonly utilized systems include the CTP score and the Barcelona Clinic Liver Cancer (BCLC) staging system. The BCLC system, which considers cancer stage, liver function, and physical condition, is widely accepted for HCC staging and treatment [30]. LT is often recommended as the primary treatment for early-stage HCC [31]. In cases where LT is not feasible, treatment options such as locoregional therapy, supportive care, and systemic chemotherapies are considered based on the patient’s condition [26]. While LT is available in Saudi Arabia, long waiting lists make it impractical for many HCC patients. Living related transplantation is gaining traction in the Kingdom, with its specific role in HCC treatment yet to be fully defined. In Japan and Saudi Arabia, an algorithm based on the CTP score of liver function is utilized, considering liver function, tumor number, and tumor size. LT is typically recommended for CTP class A or B patients, while chemotherapy becomes the preferred option in cases of extrahepatic spread. A study by Dahlan et al. in 2022 examined various treatment modalities in 108 HCC patients in Saudi Arabia, shedding light on the local treatment landscape [32]. They concluded that RFA with or without Transarterial catheter Chemoembolization (TACE) gives better prognosis with a 45% recurrence-free rate. Targeted molecular therapy also made good progress over the last five years. Sorafenib is a safe and efficient drug, traditionally indicated in HCC with BCLC stage C or B, to prolong survival, however, its use does not provide a complete cure [33,34]. Another first-line drug recommended by recently updated guidelines is lenvatinib [34]. Lenvatinib has a survival benefit for HBV-related HCC. However, the use of lenvatinib was not shown a strong beneficial effect compared to sorafenib [35,36]. Adjuvant therapies are also given in many cancer institutions for HCC patients to delay the disease progression while patients are waiting for LT.

Our current study investigates the trends and the pattern of HCC in Madinah City of Saudi Arabia and the effect of TNM staging, AFP level, and CTP on cancer progression and patients’ survival.

## Materials and Methods

A retrospective cohort review and analysis were conducted on 43 HCC patients, treated with different modalities, at single oncology center in King Fahad Hospital in Madinah city (Saudi Arabia) from 2015 to 2020. The study was conducted according to the guidelines and the Declaration of Helsinki and was approved by from the Institutional Review Board of the General Directorate of Health Affairs in Madinah. Informed consent is not required in this study. All patients had to fulfill one of the following criteria: (a) a liver lesion reported as definitive HCC on dynamic contrast-enhanced CT and/or MRI using the Liver Imaging Reporting and Data System (LI-RADS 5), and/or (b) a tissue biopsy-confirmed diagnosis of HCC. The primary endpoint of this study was to explore the trends of HCC and explore association between TNM staging, AFP and CTP on the survivals of viral and non-viral HCC patients.

Patients’ data were collected from the hospital records and tumor board charts. The data included the age onset during diagnosis, sex, nationality, associated chronic comorbidities, history of alcoholism, presenting symptoms, and the performance status was assessed by using the European Cooperative Oncology Group score (ECOG). All patients were stratified into two groups: Group (a) HCC with viral cause, and Group (b) HCC with non-viral cause. The incidence of liver cirrhosis and its severity in both groups were estimated and assessed using the CTP score classification. Serum AFP levels were also assessed in both groups. In term of histopathological classification, TNM (T: tumor size; N: lymph node extension, M: metastasis) system was used for HCC staging. Other than surgical options, additional treatment options delivered to the patients were also retrieved from hospital records, which included local therapy, supportive care, or systemic treatments. The BCLC staging system was also used in disease management.

The demographic data of all patients in both groups have been compared to the existence of liver cirrhosis, AFP levels, TNM staging, and CTP classification. The survival rate *vs*. mortality rate were estimated based on patient’s cancer progression, date of patient’s death, and the patient’s loss for follow-up. Patients lost their follow-up as their disease status have reached into a palliative stage, so they preferred to stay home. For those who their date of death has been determined, mortality or survival rate has been calculated from the date of the cancer diagnosis to the date of patient death. The time between the diagnosis of HCC to the end of December 2019 defined the survival.

### Statistical analysis

Categorical data were presented as frequency and percentage, while numerical data, following normality testing, were reported as mean ± SD or median and interquartile range (IQR). Group comparisons for categorical data were conducted using the Chi-square test or Z test, and for numerical data, independent *t*-tests or Mann-Whitney tests were employed. The probability of overall survival (OS) was assessed using Kaplan-Meier curves (KMC) and the log-rank test. Statistical significance was set at a *p*-value of less than 0.05. Data analysis for the current study was performed using SPSS version 26 (IBM, Armonk, New York, USA).

## Results

A total of 43 HCC patients were included in this study. In about 20% of patients, HCC has been detected without a clinical or radiological history of chronic liver disease (CLD). The mean patients’ age at presentation of all HCC cases was 66.8 ± 10.6 years. The majority of these patients were male (77%), with a male to female ratio of 3.3:1. About 91% (n = 39) of the cases were Saudi (Table 1).

**Table 1:** Sociodemographic and clinical characteristics of HCC patients in this study

	All patients (n = 43)	Viral (n = 22)	Non-viral (n =21)	Test	*p*-value
**Age (years)**					
Mean ± SD	66.84 ± 10.59	67.27 ± 10.66	66.38 ± 10.77	*t* = −0.27	0.791
<60 years	9 (20.9)	5 (22.7)	4 (19)	χ^2^ = 0.088	0.767
≥60 years	34 (79.1)	17 (77.3)	17 (81)		
**Sex**: **n (%)**					
Male	33 (76.7)	17 (77.3)	16 (76.2)	χ^2^ = 0.007	0.933
Female	10 (23.3)	5 (22.7)	5 (23.8)		
**Nationality: n (%)**					
Saudi	39 (90.7)	20 (90.9)	19 (90.5)	χ^2^ = 0.002	0.961
Non-Saudi	4 (9.3)	2 (9.1)	2 (9.5)		
**Comorbidities: n (%)**					
Diabetes mellitus	16 (37.2)	4 (18.2)	12 (57.1)	Z = −2.64	0.008*
Hypertension	16 (37.2)	7 (31.8)	9 (42.9)	Z = −0.75	0.454
Stroke	3 (7.0)	0 (0)	3 (14.3)	Z = −1.84	0.066
Chronic kidney disease	2 (4.7)	2 (9.1)	0 (0)	Z = 1.42	0.157
Cardiomyopathy	1 (2.3)	1 (4.5)	0 (0)	Z = 0.99	0.323
Thyroid disease	1 (2.3)	0 (0)	1 (4.8)	Z = −1.04	0.311
Bronchial asthma	1 (2.3)	1 (4.5)	0 (0)	Z = 0.99	0.323
**Alcohol consumption: n (%)**					
Yes	1 (2.3)	1 (4.5)	0 (0)	χ^2^ = 0.977	0.323
No	42 (97.7)	21 (95.5)	21 (100)		
**ECOG Performance Status: n (%)**					
0	31 (72.1)	17 (77.3)	14 (66.7)	χ^2^ = 3.56	0.314
1	7 (16.3)	2 (9.1)	5 (23.8)		
2	4 (9.3)	3 (13.6)	1 (4.8)		
3	1 (2.3)	0 (0)	1 (4.8)		
**Clinical presentation: n (%)**					
Hepatic encephalopathy	7 (16.3)	4 (18.2)	3 (14.3)	Z = 0.35	0.631
Abdominal pain	14 (32.6)	7 (31.8)	7 (33.3)	Z = −0.11	0.916
Jaundice	6 (14.0)	2 (9.1)	4 (19)	Z = −0.94	0.346
Hematemesis	4 (9.3)	2 (9.1)	2 (9.5)	Z = −0.05	0.961
Abdominal distension	2 (4.7)	0 (0)	2 (9.5)	Z = −1.48	0.138
Leg edema	2 (4.7)	0 (0)	2 (9.5)	Z = −1.48	0.138
Cough and hemoptysis	1 (2.3)	0 (0)	1 (4.8)	Z = −1.04	0.3
**Child-Turcotte Pugh score: n (%)**					
A	18 (41.9)	9 (40.9)	9 (42.9)	χ^2^ = 1	0.606
B	13 (30.2)	8 (36.4)	5 (23.8)		
C	12 (27.9)	5 (22.7)	7 (33.3)		
**Cirrhosis: n (%)**					
Yes	34 (79.1)	22 (100)	12 (57.1)	χ^2^ = 11.92	<0.001*
No	9 (20.9)	0 (0)	9 (42.9)		
**Portal vein thrombosis: n (%)**					
Yes	10 (23.3)	4 (18.2)	6 (28.6)	χ^2^ = 0.65	0.42
No	33 (76.7)	18 (81.8)	15 (71.4)		
**AFP levels (ng/mL)**					
Median (IQR)	131 (3.5–1549)	124 (3–2437)	512 (4–1100)	U = 219	0.77
<400 ng/mL	23 (53.5)	13 (59.1)	10 (47.6)	χ^2^ = 0.568	0.451
≥400 ng/mL	20 (46.5)	9 (40.9)	11 (52.4)		

Note: ECOG: Eastern Cooperative Oncology Group, IQR: inter-quartile range, *t*: independent *t*-test, χ^2^: Chi-square test, Z: Z-test for proportion, U: Mann-Whitney test, *: Statistically significant.

All patients were stratified into two groups: (a) HCC with viral cause (n = 22, 51%), and (b) HCC with non-viral cause (n = 21, 49%) (Tables 1 and 2).

**Table 2:** TNM staging, treatment and mortality rate in HCC patients in this study

	All patients (n = 43)	Viral (n = 22)	Non-viral (n = 21)	Test	*p*-value
**Tumor size (cm)**					
Mean ± SD	7.68 ± 4.27	6.54 ± 3.44	8.88 ± 4.78	*t* = 1.85	0.072
<5 cm	15 (34.9)	9 (40.9)	6 (28.6)	χ^2^ = 0.72	0.396
≥5 cm	28 (65.1)	13 (59.1)	15 (71.4)		
**Tumor number: n (%)**					
Single	15 (34.9)	5 (22.7)	10 (47.6)	χ^2^ = 2.93	0.087
Multiple	28 (65.1)	17 (77.3)	11 (52.4)		
**T stage: n (%)**					
1	5 (11.6)	2 (9.1)	3 (14.3)	χ^2^ = 2.37	0.498
2	15 (34.9)	10 (45.5)	5 (23.8)		
3	6 (14)	3 (13.6)	3 (14.3)		
4	17 (39.5)	7 (31.8)	10 (47.6)		
**N stage: n (%)**					
0	27 (62.8)	14 (63.6)	13 (61.9)	χ^2^ = 0.014	0.907
1	16 (37.2)	8 (36.4)	8 (38.1)		
**M stage: n (%)**					
0	28 (65.1)	14 (63.6)	14 (66.7)	χ^2^ = 0.043	0.835
1	15 (34.9)	8 (36.4)	7 (33.3)		
**Site of metastasis: n (%)**					
Adrenal	3 (7.0)	1 (4.5)	2 (9.5)	Z = −0.64	0.522
Lung	7 (16.3)	4 (18.2)	3 (14.3)	Z = 0.35	0.729
Peritoneal	4 (9.3)	3 (13.6)	1 (4.8)	Z = 1	0.317
Omental	2 (4.7)	2 (9.1)	0 (0)	Z = 1.42	0.157
Pleura	2 (4.7)	1 (4.5)	1 (4.8)	Z = −0.03	0.973
Bone	2 (4.7)	1 (4.5)	1 (4.8)	Z = −0.03	0.973
**Clinical stage: n (%)**					
I	5 (11.6)	2 (9.1)	3 (14.3)	χ^2^ = 2.66	0.447
II	13 (30.2)	9 (40.9)	4 (19)		
III	8 (18.6)	3 (13.6)	5 (23.8)		
IV	17 (39.5)	8 (36.4)	9 (42.9)		
**Initial treatment: n (%)**					
BSC	18 (41.9)	9 (40.9)	9 (42.9)	χ^2^ = 4.71	0.695
Lenvatinib	1 (2.3)	0 (0)	1 (4.8)		
Nivolumab	1 (2.3)	1 (4.5)	0 (0)		
RFA	1 (2.3)	1 (4.5)	0 (0)		
Sorafinib	15 (34.9)	7 (31.8)	8 (38.1)		
TACE	3 (7.0)	1 (4.5)	2 (9.5)		
TACE and TARE	1 (2.3)	1 (4.5)	0 (0)		
**Mortality: n (%)**					
Yes	17 (39.5)	10 (45.5)	7 (33.3)	χ^2^ = 0.66	0.416
No	26 (60.5)	12 (54.5)	14 (66.7)		

Note: *t*: independent *t*-test, χ^2^: Chi-square test, Z: Z-test for proportion, U: Mann-Whitney test.

The liver cirrhosis was diagnosed in 79% (n = 34) of all HCC patients, in which viral etiology contributed to 65% (n = 22/43) of HCC (Table 1). Among HCC patients due to viral cause, 55% were due to HBV infection and 45% due to HCV infection. Most of viral-HCC patients were above 60 years, and male predominance. There were insignificant statistical differences in age and sex among viral and non-viral HCC patients, respectively (*p*-value = 0.791, *p*-value = 0.933). These differences in age and sex were also insignificantly differed in OS among viral and non-viral HCC patients (*p*-value > 0.05). Chronic diabetes was the only significant chronic morbidity disease that statistically differed between viral-HCC (n = 4) and non-viral HCC (n = 12) (*p*-value = 0.008). Nonetheless, diabetes was frequently occurred in patients with viral-HCC (Table 1). Other comorbidities such as hypertension, stroke, kidney disease, and cardiomyopathies were not significantly differed between the two HCC variants (*p*-value > 0.05). The relationship between chronic alcohol intake and occurrence of HCC was also statistically insignificant (*p*-value = 0.323) because alcoholism is uncommon habit in Saudi Arabia.

**Figure 1:**
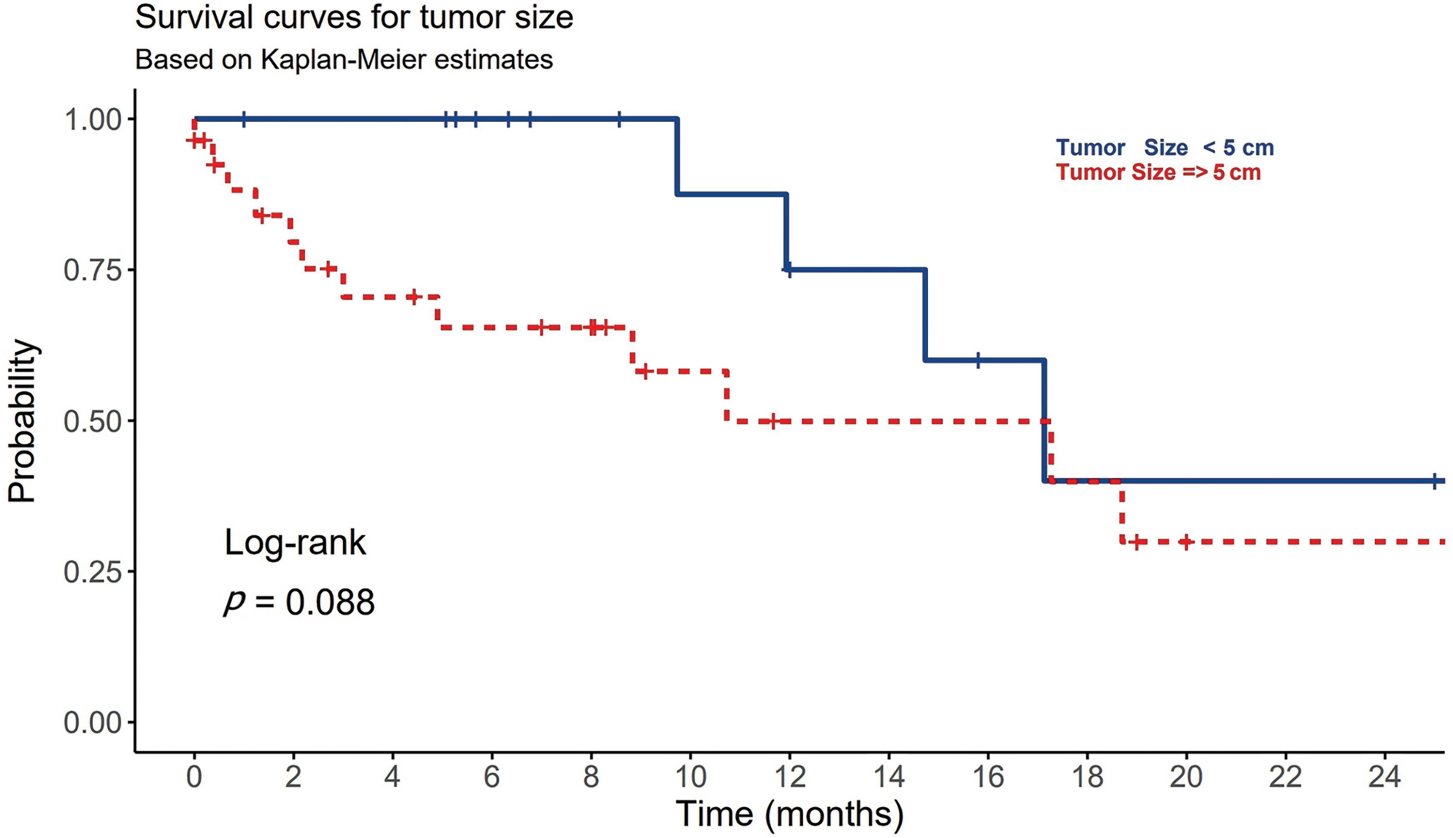
The association between tumor size (T) and the overall survival in patients with HCC.

At time of diagnosis, 53% of HCC patients were symptomatic. The most common presenting symptoms were abdominal pain (32.6%), hepatic encephalopathy (16.3%) and upper gastrointestinal tract bleeding (9.3%). Other symptoms such as jaundice, hematemesis, abdominal distention, and leg edema totally represented 14% of all patients’ symptoms. Single patient presented with hemoptysis and cough which was proven to be a lung metastasis. None of these reported symptoms were significantly differed among viral and non-viral HCC (*p*-value > 0.05) (Table 1). Ten patients (23%) in the cohort underwent liver biopsy, and in 90% of them the biopsy confirmed a diagnosis of HCC histologically, whereas it was non-conclusive in the other 10%. Upon reviewing patients’ pathological reports, about 65% (n = 28) of HCC patients were detected to have more than 5 cm tumor size during the diagnosis, with no statistically significant differences observed between the viral (n = 13) and non-viral HCC (n = 15) variants (*p*-value = 0.072) (Table 2). Most these tumors were multiple lesions (65%). However, a significant statistical difference in OS was noted for the tumor size among all HCC patients regardless their underlying variant (*p*-value = 0.027) (Table 3). HCC patients with tumor size more than 5 cm significantly negatively associated with OS (Fig. 1).

**Table 3:** The significant relationship between HCC underlying type, tumor size and AFP serum levels with the overall survival (OS)

		OS				
		Mean	Std. Error	95% confidence interval		*p*-value
				Lower bound	Upper bound	
**Underlying cause**	**Viral**	14.712	2.804	9.216	20.208	0.008*
	**Non-viral**	16.368	2.342	11.777	20.960	
**Tumor size**	**<5 cm**	20.343	3.224	14.024	26.662	0.027*
	**>5 cm**	13.791	2.529	8.835	18.748	
**AFP levels**	**<400 ng/mL**	22.264	2.635	17.100	27.428	0.021*
	**>400 ng/mL**	9.697	1.733	6.300	13.094	

Note: *: Statistically significant.

Stage IV was the most frequent HCC staging (n = 17, 39.5%) followed by stage II (n = 13, 30.2%) (Table 2). However, there was statistically insignificant difference in TNM staging among all HCC patients regardless their underlying variant (*p*-value = 0.447). The most frequent site of metastasis was the lung (16.3%) and peritoneum (9.3%) followed by adrenal gland (7%) and omentum (4.7%), pleura (4.7%) and bones (4.7%) (Table 2). Insignificant statistical difference in the site of metastasis was observed among viral and non-viral HCC patients (*p*-value > 0.05). Portal vein thrombosis was found in approximately 77% of HCC patients with no significant difference observed between viral and non-viral variants (*p*-value = 0.42) (Table 1).

**Figure 2:**
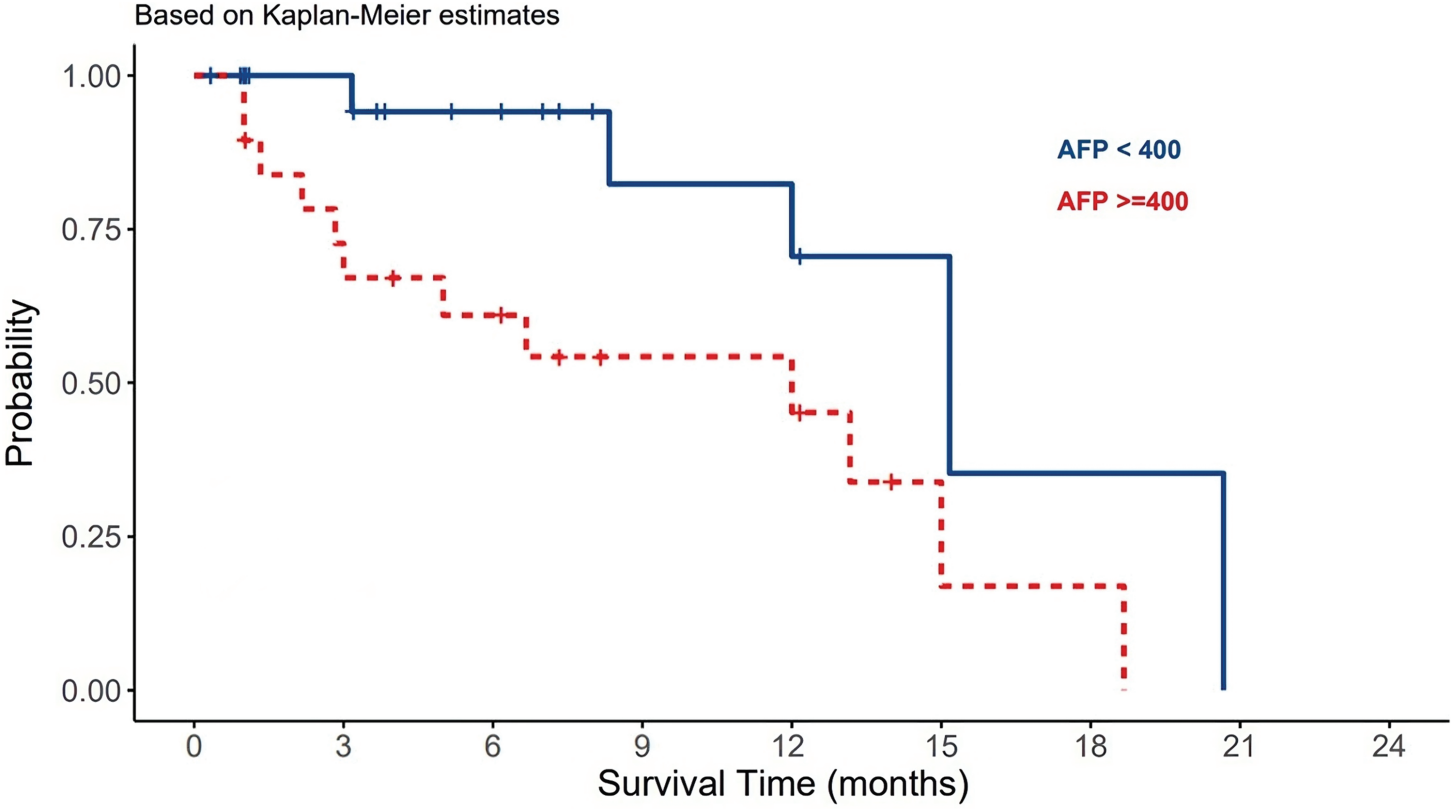
The effect of AFP serum levels on the survival of HCC patients.

The median AFP serum levels in patients with viral-HCC were 124 ng/ml compared to non-viral HCC patients (Table 1). AFP levels were found to be normal in 29% of the patients, and greater than 400 ng/ml in 45% of the patients. Statistically, there was insignificant differences in AFP serum levels among viral and non-viral HCC patients (*p*-value = 0.77). However, the differences were significantly observed in the OS in term of AFP serve levels (*p*-value = 0.021) (Table 3). AFP levels more than 400 ng/ml were associated worsened outcome compared to levels less than 400 ng/mL (Fig. 2).

**Figure 3:**
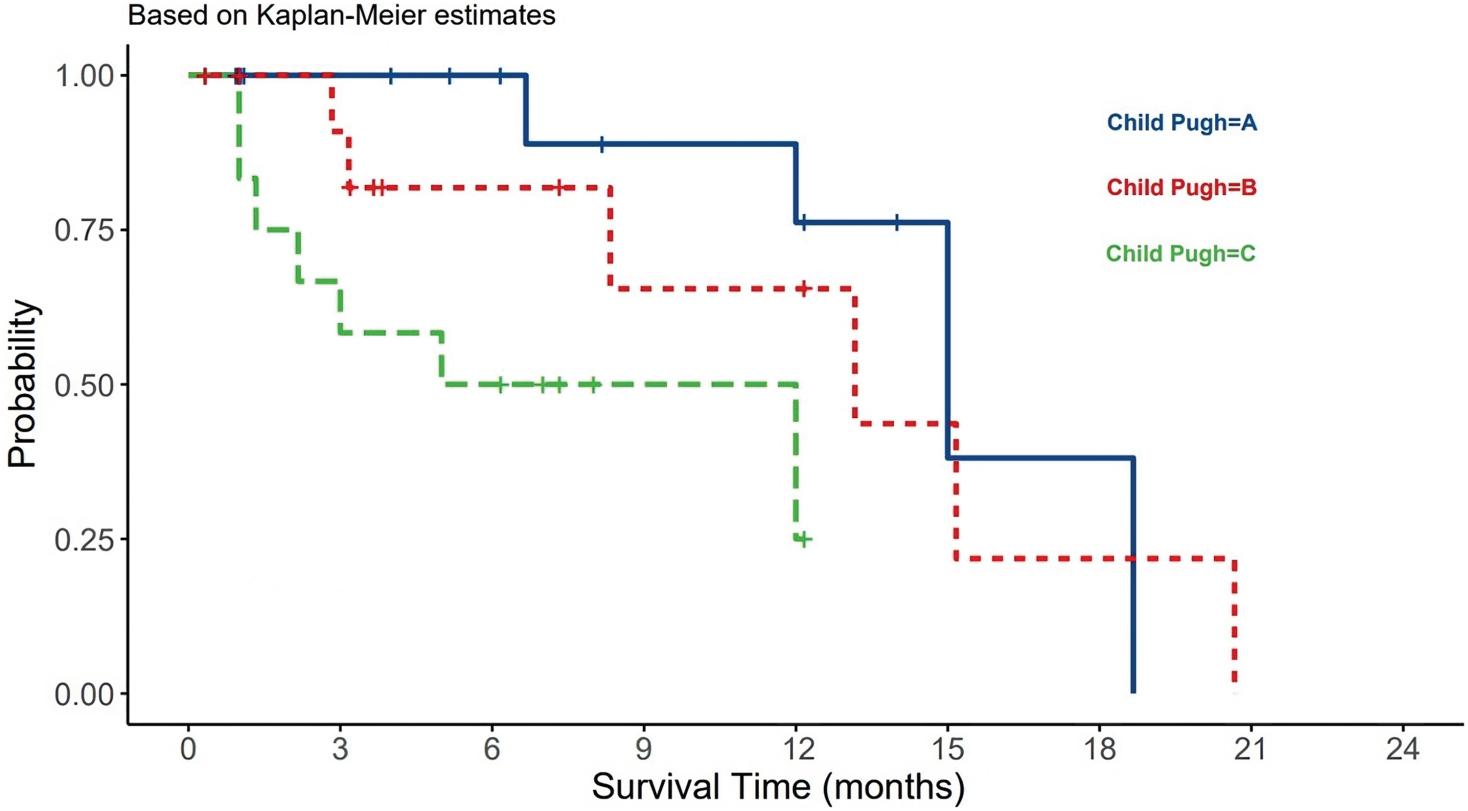
The relationship between CTP and HCC patients survival.

For the CTP score, most patients presented with a score A (42%), whereas 30% and 28% had score B or C, respectively. There was a statistically significant difference between different CPT classes and OS (*p*-value = 0.02). CPT class B had the longest survival among A and B classes (Fig. 3). On multivariate analysis using Cox regression, CTP score HR (3.309) and elevated AFP HR (6.927) were significantly associated with OS. Most of the patients had stage D (40%) or stage C (35%) HCC per the BCLC score.

**Figure 4:**
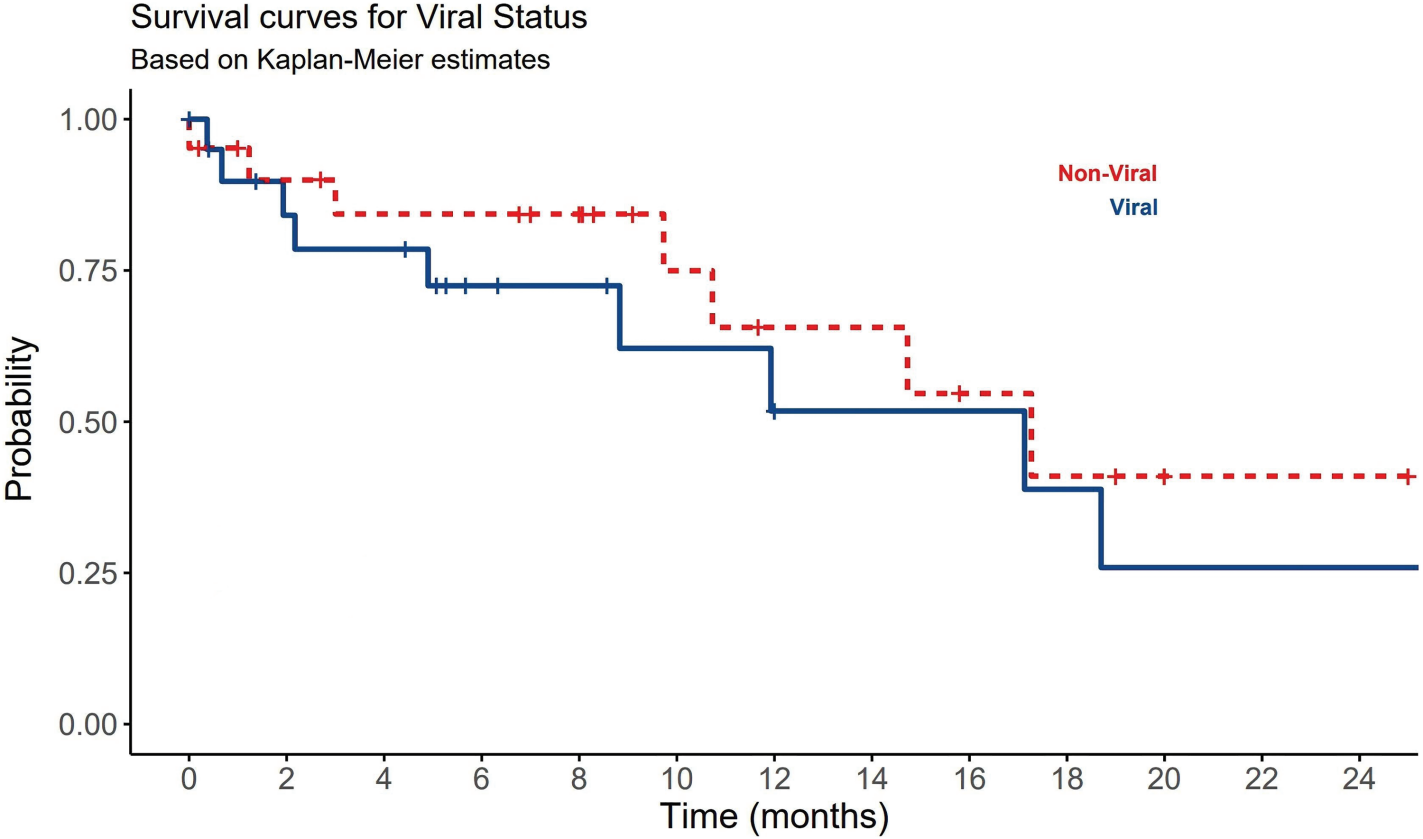
The significant impact of viral and non-viral causes of HCC on patients OS.

Best supportive care (BSC) was the most common treatment plan initially provided to 41.9% of HCC patients. Regardless of other treatment options offered to the patients and their availability, systemic therapies were only given to 22 (51%) patients (Table 2). Sorafenib, tyrosine kinase inhibitor, was the most frequent systemic therapy given to 34.9% (n = 15) of HCC patients regardless HCC underlying type. Other systemic therapies included lenvatinib, nivolumab, RFA, Transcatheter arterial chemoembolization (TACE), TACE + trans-arterial radioembolization (TARE) were also given to some patients based on their conditional status. Only 12% of HCC patients have received local ablation or chemoembolization. Systemic chemotherapies showed no statistically significant differences between viral and non-viral HCC patients (*p*-value = 0.695) (Table 2). About 39.5% (n = 17) of the HCC patients have died and the remining 60% (n = 26) of patients lost their follow-up or showed cancer progression. Although there was no significant difference between viral and non-viral HCC, a significant statistical difference in OS among viral and non-viral HCC patients was observed (*p*-value=0.008) (Table 3). Nonetheless, patients with viral-HCC survived more than patients with non-viral HCC (Fig. 4). All data results are described and summarized in Tables 1–3 and Figs. 1–4.

## Discussion

HCC is the primary cause of cancer-related mortality globally and in Saudi Arabia [1–3]. The majority of HCC cases are of viral origin, with HBV infection being the predominant cause. HBV infection contributes to over 50% of HCC cases, although regional disparities exist [5]. In Korea, approximately 70% of HCC cases are linked to HBV, while in Japan the USA, and Sweden, the percentages are 16% and 5%, respectively [37,38]. Conversely, HCV is recognized as the most significant risk factor for HCC in Western countries and among Saudi patients [29]. About 51% of the HCC patients in our study tested seropositive for HBV (55%) or HCV (45%), which was less than what has been reported by Aljumah et al and Alsawat et al studies, in which 75%–80% of HCCs were associated with viral causes [11,12]. Alswat et al reported that HCV was detected in 48% of the cases [12]. In both studies, the number of patients was larger than our study samples, which concludes more logically. The change in the pattern of viral variation for HCC in Saudi patients has been attributed to the recent implementation of HBV vaccination, while the non-viral causes in the community such as smoking, obesity, and diabetes have increased steadily. The progression from HBV or HCV to HCC may vary between populations based on different factors. Hence, not every HBV or HCV carrier develops HCC and the most probable predisposing factors for this progression are the genotype of the virus and the stage of liver disease [5]. HBV genotypes exhibit varying risks for HCC development. Genotype C carries a higher HCC risk compared to genotypes B and D [39]. The presence of liver cirrhosis is also a significant risk factor for HCC development, irrespective of the underlying cause [40,41]. The annual incidence of HCC in individuals with compensated cirrhosis is approximately 3% [42]. Interestingly, HCC was observed to occur more frequently in non-cirrhotic livers than in HBV-related cases [43]. Overall, the rate of HCC development in HCV ranges from 1% to 3% after 30 years and the incidence of HCC in HBV carriers is around 0.5% [44,45]. In our study, liver cirrhosis was diagnosed in 34 out of 43 (79%) patients with HCC (viral > non-viral). This association between liver cirrhosis and HCC was highly significant (*p*-value < 0.05), which means that most patients with liver cirrhosis have developed HCC, and viral type was more frequent than non-viral type (Table 1).

The age at the time of infection is also another factor that increases the risk of HCC progression [1]. Population above 60 years with underlying risk factors of HCC or CLD develop HCC faster than the young population except data from African countries which showed a younger mean age at the time of diagnosis [46,47]. These differences can be attributed to the patterns of risk factors found in those countries. The incidence of HBV and exposure to aflatoxin in African countries are extremely high [48]. Although the reasons for gender differences in the incidence of HCC are not fully understood, one possible factor is the protective role of estrogen through the inhibition of interleukin 6, thereby reducing liver cell injury. Another possible factor is related to high male exposure to environmental toxins [48]. In our study, the median age at diagnosis of HCC was 66 years and most of the patients were male. These results are in concordance with most local and international studies. Registered data from the Saudi Observatory Liver Disease Registry (SOLID) in the period from 2003 to 2008 reported on 366 patients, also found that the mean age of HCC was 66 years and most of the patients were male [5]. Age and sex were not predictors for survival among all HCC patients in our study (Table 1). Insignificant statistical relationships in age and sex among viral and non-viral HCC patients were also noted and did not differ in OS among both groups (*p*-value > 0.05). The risk of progression to HCC was also reported to increase with alcoholism, obesity, and NAFLD [49–51]. Our study found that chronic diabetes was significantly prevalent among patients with viral HCC (Table 1). This explains that obesity or metabolic syndrome due to long-standing diabetes plays a significant role in NASH and the progression to HCC. Progression from NASH to NASH-related HCC (2% of cases per year) has been commonly reported and was found to be influenced by many factors, including immune microenvironment [52]. Other comorbidities reported in our study such as hypertension, stroke, kidney diseases, and cardiomyopathies were not found significantly related to HCC progression for both types (Table 1). Chronic alcohol intake was also not a predisposing risk factor for the development of HCC in Saudi Patients because alcoholism is an uncommon habit in Saudi Arabia. However, heavy alcohol intake was a major risk factor for developing HCC in other different populations. A study conducted in Italy found that HCC was developed in drinkers 13 times greater than its occurrence in non-drinkers [53].

AFP is an alpha-1 globulin typically found in high levels in fetal serum, while adults have only trace amounts. According to a study conducted by Daniel et al, the sensitivity and specificity of AFP in HCC screening were reported as 40%–60% and 70%–90%, respectively. The study also highlighted a low positive predictive value of up to 50% [54]. A multi-center study on 206 cases in Saudi Arabia in 2010, to assess the diagnostic benefits of AFP in HCC, found that AFP is a poor diagnostic value for HCC [55]. An AFP > 100 ng/ml has a high degree of specificity and may be used as a confirmatory test. Our study is the second research that assessed AFP in HCC. AFP levels were found to be normal in 29% of the HCC patients, and greater than 400 ng/ml in 45% of the patients. However, no significant differences were noted in AFP levels to segregate viral from non-viral causes (Table 1). On the other hand, AFP > 400 ng/ml was found to be associated with a poor outcome among HCC patients (Fig. 2). Because AFP can be elevated in patients with cholangiocarcinoma or metastatic colon cancer, its diagnostic use is less specific and beneficial [56].

Radiological findings and liver biopsy are considered diagnostically more useful than AFP testing. However, obtaining a tissue biopsy for HCC is not a mandatory methodology in cases of HCC. Tissue biopsy is commonly required when liver imaging is atypical, or the lesion size is more than 1 cm [5]. The diagnostic accuracy of tissue biopsy was estimated to be 97% [5]. In our research, 90% of biopsied patients confirmed HCC, and in 10% of the remaining cases, the biopsy was inconclusive. Those inconclusive results were due to the small size of the lesions. Alternatively, dynamic contrast CT/MR imaging is currently considered the best non-invasive method to diagnose HCC and to determine the clinical staging of the disease [5]. In our study, about 65% of HCC patients were detected to have more than 5 cm tumor size during the diagnosis. Those patients showed poor OS (Fig. 1). For a lesion smaller than 1 cm, a repeated US is recommended in 3 to 6 months. If the lesion is shown to be growing, then further investigations are required. The most common staging system is Barcelona Clinic Liver Cancer (BCLC). This scoring system is very clinically oriented. BCLC includes variables associated with anatomical stage, liver function, and patient symptoms. Each stage leads to a pathway for the selection of treatment modality [33]. Approximately, 40% of our patients presented with an advanced stage (stage D), and a minority (9%) presented in the early stages (stages 0, A), which are curable stages. This may reflect poor compliance with surveillance guidelines for HCC. Aljumah et al. reported that 10% of 172 patients enrolled in their study had stage D disease, whereas another study conducted by Alswat et al. reported that 60% of 363 studied patients had an advanced HCC [11,12].

The CTP system was designed to predict mortality in cirrhotic patients [5]. It has been designed to select patients who would benefit from elective surgery for portal decompression. CTP divides patients into three categories: A-good hepatic function, B-moderately impaired hepatic function, and C-advanced hepatic dysfunction. In our study, 42% of HCC patients presented with a score of A. Class A is compensated disease with 100% 1-year OS and 80% 2-year survival [57]. However, we found no significant differences in CTP scores between viral and non-viral HCC but a significant difference in OS was found among all classes, in which CTP class B had the longest survival (Fig. 3). On multivariate analysis using Cox regression, CTP and AFP were significantly associated with OS (*p*-value < 0.05).

Although LT is considered the best treatment option for HCC, LT in Saudi Arabia requires long waiting times. Alternatively, living-related transplantation, locoregional therapy, BSL, and chemotherapies are temporary plans used to shrink the tumor size and improve patient survival. LT and locoregional therapies are usually indicated in the early stages (BCLC O, A, B, and CPT A, B). RFA is one of the common alternative plans that increases OS for up to 3 years [58]. Patients with an extended disease, poor hepatic reserve, or coexistent morbidity have been treated using chemoembolization. TACE or TAR has shown a 95% 5-year survival rate in this context [59]. While tumor resection can remove the visible portion of cancer, it is not as effective as LT in ensuring the removal of non-visible tumors and microscopic satellite lesions. The key principles of liver resection in cirrhotic patients include parenchymal preservation, minimal blood loss, and a negative resection margin of at least 1 cm. RFA is commonly available in tertiary care centers in Saudi Arabia but not in private centers. Studies, such as the one conducted by Dahlan et al., have shown that the combination of TACE and RFA or RFA alone can lead to better outcomes, with a recurrence-free rate of up to 50% [32].

Systemic chemotherapies have not yielded promising results in the treatment of HCC. Sorafenib has emerged as the most effective therapy for improving the survival of HCC patients. It is particularly recommended for patients in advanced stages (BCLC C or CPT A and B). Sorafenib is a tyrosine kinase inhibitor known to target Raf-1, B-Raf, VEGFR2, PDGFR, and c-Kit receptors [60]. The use of sorafenib as an adjuvant therapy, either in combination with other modalities like TACE or RFA or as a precursor to LT, is currently under extensive research. Because of the often-advanced stage at presentation, the most common treatment in our study was BSL, followed by other tyrosine kinase inhibitors. All treatments did not show any significant impact on OS. However, the treatment plans including LT were not determined well by all patients. RFA, TACE, and TAR are different pre-surgical methods in the treatment of HCC. The most robust predictors for survival in our study were underlying HCC cause, AFP, and tumor size. This is compatible with previous local and international studies [32,60]. Being having non-viral etiology, a tumor size > 5 cm, an AFP > 400 ng/mL, and a CTP score class C, were all significantly negatively associated with OS (Figs. 1–4) (Tables 1–3).

## Conclusions

The pattern of HCC has changed in Saudi patients, and non-viral risk factors have become more prevalent. Most HCC in non-cirrhotic livers was related to non-viral etiologies. Substantial numbers of our patients are still present in the advanced stages and the reason for this observation needs to be explored in future studies. This is important to inform health authority decision-makers regarding strategies aiming to improve early HCC diagnosis and intervention.

## Data Availability

The datasets generated during and/or analyzed during the current study are available from the corresponding author (NM) on reasonable request.
